# Letter from the Editor in Chief

**DOI:** 10.19102/icrm.2026.17013

**Published:** 2026-01-15

**Authors:** Devi Nair


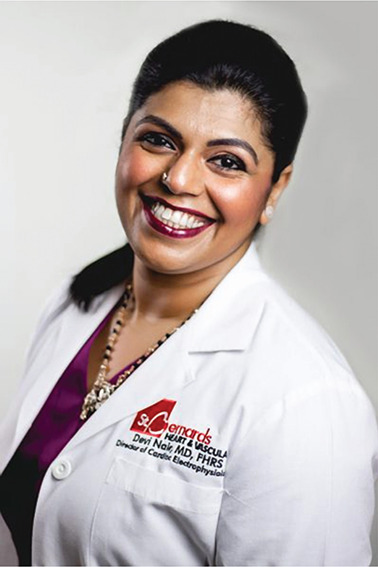


Dear Colleagues,

As we begin a new year, I would like to wish you all a happy, healthy, and fulfilling 2026. January offers a natural moment to pause, both to take stock of how far our field has come and to consider the questions that will shape the year ahead. The pace of progress in cardiac electrophysiology remains extraordinary, and 2025 was a year in which innovation increasingly transitioned from promise to practice.

Looking back, one of the clearest themes of 2025 was maturation. Technologies that only recently felt early or exploratory became more broadly adopted, better defined by clinical evidence, and increasingly standardized in workflow. We saw continued refinement in ablation techniques and energy delivery, along with an expanding focus on procedural efficiency, safety, and lesion durability. In parallel, advances in mapping and imaging helped us move beyond “one-size-fits-all” strategies toward more individualized procedural planning, particularly in patients with persistent atrial fibrillation and complex substrates.

Ablation remained a major center of gravity. Pulsed field ablation (PFA) continued to generate momentum, not simply as a novel technology but also as an evolving platform, and 2025 was a year in which the conversation surrounding it became more nuanced. Beyond initial safety enthusiasm, the field increasingly focused on durability: how well lesions hold over time; what patterns of reconnection emerge; and what procedural variables, energy delivery, catheter contact, waveform choices, and lesion set strategies most influence long-term success. Alongside durability, workflow became a defining topic. Many labs moved from early adoption to optimization, refining case flow, anesthesia strategy, intraprocedural verification, and post-procedure pathways to fully realize the efficiency potential of PFA while maintaining rigorous endpoints.

Importantly, 2025 also brought healthy, necessary discussion around PFA-specific observations and adverse-event signals. As experience broadened, operators shared practical approaches to phenomena such as coronary artery spasm, with increasing recognition of proximity-related effects versus more generalized vasoreactivity. In parallel, the community paid close attention to reports and mechanistic hypotheses related to hemolysis in certain settings, prompting deeper consideration of procedural parameters, patient selection, and best practices to minimize risk. These discussions reflect a field doing what it does best: moving beyond novelty to responsible refinement, strengthening safety, clarifying mechanisms, and standardizing technique through collective learning.

Another notable shift in 2025 was the continued evolution of care delivery models. With accumulating experience, stronger safety frameworks, and increasingly efficient workflows, discussions about extending atrial fibrillation ablation beyond the traditional hospital setting accelerated. Importantly, however, broad practice change had not yet occurred. Rather, the key milestone came late in the year, when approval pathways for performing AF ablation in ambulatory surgery centers emerged, setting the stage for future adoption. This development reflects not only advances in technology but also the maturation of peri-procedural pathways—the proper patient-selection choices, anesthesia approaches, and post-procedure monitoring needed to maintain safety while improving access and operational efficiency.

Pacing and device therapy also experienced meaningful evolution in 2025, with physiologic pacing increasingly becoming a clinical expectation rather than a niche technique and with the field expanding its definition of what “physiologic therapy” can be. Conduction system pacing (CSP) continued to grow through His-bundle pacing and left bundle branch area pacing, supported by broader operator experience and an expanding evidence base. At the same time, the conversation moved beyond where to pace to how best to deliver comprehensive therapy: the emerging potential of CSP-capable implantable cardioverter-defibrillator lead strategies, the promise of integrating defibrillation with physiologic activation, and early explorations into whether CSP approaches can be paired more seamlessly with device-based heart failure management.

2025 also offered a glimpse of the next frontier through first-in-human work in leadless physiologic pacing concepts, pointing toward a future where conduction system capture and modular platforms may reduce the long-term burden of leads and pockets, particularly in younger patients and those at high risk for complications. In parallel, neuromodulation therapies continued to evolve, reflecting renewed interest in how autonomic targets may complement ablation and device therapy in select arrhythmia and heart failure populations. Beyond pacing alone, cardiac contractility modulation continued to build its clinical footprint as an option for carefully selected patients, and trials evaluating emerging hemodynamic therapies—such as atrioventricular interval modulation approaches—highlighted the broader push toward device-based strategies aimed at improving symptoms and functional capacity in heart failure.

Importantly, 2025 also reminded us that enthusiasm must be paired with rigor. Data from late-breaking trials such as the PhysioSync study reinforced that physiologic pacing is not automatically superior in every substrate or clinical setting and that outcomes depend on patient selection, implantation technique, and the comparator being used. These results were a valuable caution against assuming a universal benefit, underscoring the need to individualize CSP strategies, define appropriate endpoints, and continue building a high-quality evidence base that guides when physiologic pacing should complement or defer to established resynchronization approaches.

Digital health and artificial intelligence (AI) also took a meaningful step forward in 2025, moving from “adjacent” tools to increasingly integrated components of rhythm management. AI-driven analytics continue to enhance arrhythmia detection and triage through devices and wearables, but the most compelling evolution was procedural. In atrial fibrillation, AI-assisted mapping technologies advanced efforts to identify substrate and drivers beyond purely anatomic lesion sets. In ventricular tachycardia, AI-enabled imaging and automated scar characterization strengthened pre-procedure planning and substrate-based ablation strategies. AI-driven imaging and decision-support tools also increasingly informed left atrial appendage management, refining patient selection, procedural planning, and longitudinal follow-up as left atrial appendage therapy becomes more integrated into comprehensive atrial fibrillation care.

Yet, as these tools become more powerful, the same principle that has guided us so far applies: meaningful integration requires transparency, validation, and thoughtful clinical pathways so that technology augments expertise rather than replaces judgment. The promise of these technologies is substantial, but so too is the responsibility to ensure that new tools improve outcomes, reduce variability, and expand access rather than introduce new blind spots.

In this January issue, we bring together multiple “2025 in Review” commentaries authored by leaders and key opinion leaders across electrophysiology, offering diverse perspectives on the advances, challenges, and unresolved questions that defined the past year and will shape the direction of the field moving forward.

As we move into 2026, we do so with momentum and with a clear understanding that progress in electrophysiology is rarely linear. It comes from rigorous evidence, careful iteration, candid discussion, and a shared commitment to continually improving the lives of our patients. The Journal remains dedicated to serving as a platform for that work: for innovation, for education, and for the dialogue that turns discovery into better care.

Thank you to our authors, reviewers, and readers for your continued trust and engagement. It is a privilege to serve this community, and I look forward to another year of shared learning, collaboration, and progress.

Warm regards,


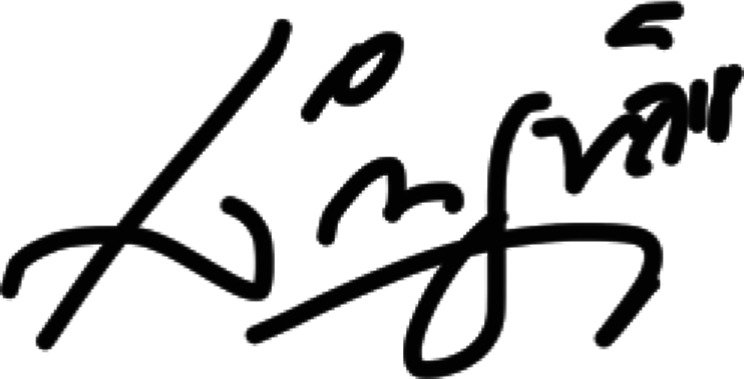


Dr. Devi Nair, md, facc, fhrs

Editor-in-Chief

*The Journal of Innovations in Cardiac Rhythm Management*

Director of the Cardiac Electrophysiology & Research,

St. Bernard’s Heart & Vascular Center, Jonesboro, AR, USA

White River Medical Center, Batesville, AR, USA

President/CEO, Arrhythmia Research Group

Clinical Adjunct Professor, University of Arkansas for Medical Sciences

Governor, Arkansas Chapter of the American College of Cardiology

drdgnair@gmail.com

